# Use of tobacco, nicotine and cannabis products among students in Switzerland

**DOI:** 10.3389/fpubh.2023.1076217

**Published:** 2023-03-29

**Authors:** Jana Affolter, Eveline Rohland, Marc Philippe, Kali Tal, Reto Auer, Julian Jakob

**Affiliations:** ^1^Department of Health Promotion and Prevention, Swiss Lung Association Aargau, Aarau, Switzerland; ^2^Sozialdienst Limmattal, Suchtpräventionsstelle der Bezirke Affoltern und Dietikon, Schlieren, Switzerland; ^3^Department of Health Promotion and Prevention, Swiss Lung Association St. Gallen - Appenzell, St. Gallen, Switzerland; ^4^Institute of Primary Health Care (BIHAM), University of Bern, Bern, Switzerland; ^5^Center for Primary Care and Public Health (Unisanté), Lausanne, Switzerland; ^6^Department of Paediatrics, University Hospital Bern, Inselspital, Bern, Switzerland

**Keywords:** tobacco, nicotine, e-cigarettes, snus, cannabis, adolescent, prevalence

## Abstract

**Introduction:**

Most people who smoke cigarettes begin in their teens and teens may also be attracted to new tobacco, nicotine, and cannabis products. We describe use prevalence among upper-secondary school students in Switzerland, including daily use, of tobacco, nicotine, and cannabis products.

**Methods:**

We invited secondary school students (age 15 to 21) in two Swiss cantons to take an online survey between October 2021 and February 2022. The survey collected demographic information and asked how frequently they used tobacco products (cigarettes in commercial packages, self-rolled cigarettes, hookahs, pipes, cigars and cigarillos, tobacco heating systems, snus, snuff), non-tobacco nicotine products (nicotine pouches, e-cigarettes with and without nicotine), and cannabis products (smoking with and without tobacco, cannabis vaping). Answers were scored on a Likert scale (no use in past month, less than weekly, weekly but not daily, daily use, prefer not to say), then tabulated and reported as descriptive statistics.

**Results:**

Of 32,614 students in the schools we contacted, 9,515 (29.2%) completed the survey; 49.5% identified as female and 48.4% as male; 9.5% were under 16, 47% were 16–17, 27.5% were 18–19, and 16% were over 19. Reported daily use was most frequent for tobacco cigarettes in commercial packages (14.2%), snus (4.1%) and cannabis smoking with tobacco (3.6%). Most participants (54.8%) reported they had used at least one product at least once within the last month.

**Conclusion:**

Students who used a product were most likely to smoke cigarettes, but many regularly used new tobacco, nicotine and cannabis products, though use frequency varies.

## 1. Introduction

Tobacco cigarettes have long been popular with adolescents, and many are also using new tobacco, nicotine and cannabis products. While alternatives like e-cigarettes (with and without nicotine), snus, snuff, and nicotine pouches may reduce the burden of disease linked to tobacco smoking (1, 2), there is concern that these new products may also threaten public health. They may increase the number of young people addicted to nicotine (3–6) or introduce risks to younger populations as the age of smoking initiation declines in Europe (7, 8). The potential risks posed by tobacco, nicotine and cannabis products to individuals are an increasing subject of study, but more information about the prevalence of product is needed to estimate health risks at the population level.

Without prevalence data on substance use, policy makers cannot devise and prioritize prevention programs or write effective legislation (5, 8–11). In countries like Switzerland, which are “tobacco industry friendly,” (12), health and prevention professionals need this data to persuade political decision makers to adopt policies that protect vulnerable populations. But few studies have described usage of these new tobacco, nicotine, and cannabis products in adolescents in Europe (8, 13–17) and we know of no study that assessed prevalence and frequency of use across the variety of products. We thus set out to ascertain prevalence and frequency of use of a broad range of tobacco, nicotine, and cannabis products by surveying students in Switzerland and determining associations between product use and socio-demographic factors such as age, gender, and school type.

## 2. Methods

### 2.1. Participants

Students in two Swiss cantons who completed compulsory schooling and attend a secondary school (vocational schools, academic high school, or in their 10th grade interim year, usually at 15 years of age and older) were invited to take an online survey between October 2021 and February 2022.

In Switzerland, compulsory schooling comprises two years of kindergarten and 9 years of school. After completing compulsory education, adolescents may go to vocational schools or enter academic high schools (gymnasium). Adolescents who attend basic vocational schools take classes from 1 to 3 days per week and learn the theory necessary to support their career internships (e.g., traditional manual jobs). Adolescents who attend higher vocational schools learn more theory and spend more time in class besides their career internship (e.g., nursing, electrician), which allows them to remain in their job or attend university after passing additional exams. Adolescents who qualify for the academic track right away may attend academic high schools (gymnasium) and usually enroll in university directly after high school. The 10^th^ school grade is an interim year for students who finished compulsory schooling and want to deepen their general knowledge while preparing for a higher vocational school.

We contacted all secondary schools in two Swiss cantons. The representatives delegated by each institution decided how strongly they would promote the survey at their school. To motivate administrators to participate, we offered to individually evaluate the substance use patterns at each school.

The school principal's office invited students to participate by email, or teachers directly invited students in their class to take the survey. To protect students' sense of privacy and reduce selection bias, students accessed the survey on their own smartphone or laptop by scanning a QR code or typing in a URL.

### 2.2. Measurements and statistics

The survey was brief, taking about 5 min to complete. We asked participants for demographic information (gender, age) and type of school. They filled out information about their use of three categories of product within the last month: tobacco products (tobacco cigarettes in commercial packages, self-rolled tobacco cigarettes, hookahs, pipes, cigars and cigarillos, tobacco heating products, snus, snuff); non-tobacco nicotine products (nicotine pouches, e-cigarettes with nicotine, e-cigarettes without nicotine); and, cannabis products (cannabis smoking with and without tobacco mulling, cannabis vaping). They reported their answers on a Likert scale (no use in the past month, less than weekly but at least monthly, weekly but not daily, daily use, prefer not to say). Since biologically verifying self-declared exposure would have lengthened the survey and required signed informed consent, we did not include it in the experimental design. We tabulated the data and generated descriptive statistics, using IBM SPSS Statistics 28.0 for all data analyses.

To more easily associate these varied products with demographic data and compare use, we created separated products into four categories: (1) tobacco burning products (tobacco cigarettes in commercial packages, self-rolled tobacco cigarettes, hookahs, pipes, cigars, and cigarillos, tobacco heating products), (2) e-cigarettes (with and without nicotine); (3) oral tobacco products (snus, snuff, nicotine pouches); and, (4) cannabis products (cannabis smoking with tobacco mulling, cannabis smoking without tobacco mulling, cannabis vaping). If students used several products from a single category, we counted the product they used most frequently. To reduce the effects of possible gender-, age-, and education-based differences in product use across the four categories, we performed Chi^2^-tests on weekly or more frequent use (sum of “daily” and “weekly but not daily” use). We compared weekly or more frequent use of multiple products in these four categories. We considered *p* < 0.05 to be statistically significant.

## 3. Results

Of the 32,641 students in the schools we contacted, 10,036 opened the survey. We excluded students who did not answer questions about substance use (*n* = 478) and students who claimed they used all products daily (*n* = 43), finally including 9,515 students in our analyses (29.2%). Student response rate was 29.0% for basic vocational and higher vocational school students, 29.6% for high school students, and 48.9% for students in their 10^th^ grade interim year.

**Figure 1:**
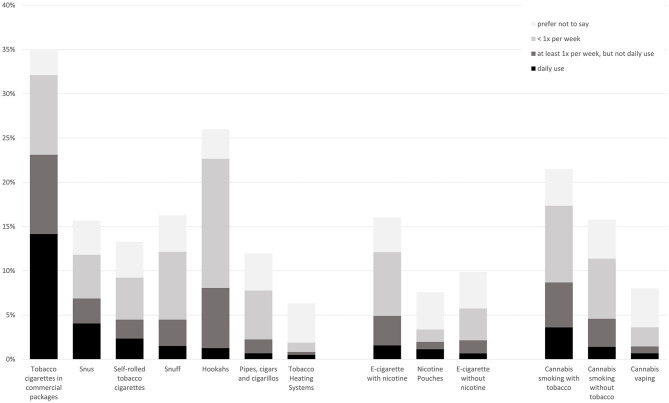
Proportions students who reported using tobacco, nicotine, or cannabis products within the last month (n = 9515), ordered by category and proportion of reported daily use.

We found 4,712 participants identified as women (49.5%), 5,382 claimed they were under 18 (56.5%), and 6,276 (66%) attended a vocational school (see Table 1); 43.6% claimed they used none of these products within in the last month. Reported daily use was most frequent for commercially packaged tobacco cigarettes (14.2%), snus (4.1%), and cannabis mulled with tobacco (3.6%). Highest weekly use was reported for commercially packaged tobacco cigarettes (23.1%), cannabis mulled with tobacco (8.7%), hookahs (8.1%), snus (6.9%), and e-cigarettes with nicotine (4.9%). Highest monthly usage was reported for commercially packaged tobacco cigarettes (32.1%), hookahs (22.7%) and cannabis mulled with tobacco (17.4%) (See Figure 1 for proportions and frequency of use).

**Table 1:** Demographic data of participants (*n* = 9,515).

**Gender**	***n***	**%**
Women	4,712	49.5
Men	4,606	48.4
Other	197	2.1
**Age**		
<16	910	9.6
16–17	4,472	47.0
18–19	2,613	27.5
>19	1,520	16.0
**Education**		
Basic vocational school^*^	6,276	66.0
Higher vocational school^**^	1,309	13.8
High school^***^	1,695	17.8
10^th^ school grade^****^	235	2.5

* Adolescents who attend basic vocational schools take classes from 1 to 3 days per week and learn the theory necessary to support their career internships (e.g., traditional manual jobs).

** Adolescents who attend higher vocational schools learn more theory and spend more time in class besides their career internship (e.g., nursing, electrician), which allows them to remain in their job or attend university after passing additional exams.

*** Adolescents who qualify for the academic track immediately may attend academic high schools (called gymnasium) and usually enroll in university directly after high school.

**** The 10^th^ school grade is an interim school year for students who finished compulsory schooling and want to deepen their general knowledge in preparation for higher vocational school.

### 3.1. Single and multiple use

We compared single and multiple use in students who reported at least weekly use: 3,659 students used products in at least one category at least once a week. Of these, 2,799 people used tobacco-burning products, 1,003 used oral products, 587 used e-cigarettes, and 936 used cannabis products. Most students (60.3%) used products from a single category (single use), 31.9% used products from two categories (dual use), and 7.7% used products from three or four categories at least weekly (see Table 2 for summaries of single and multiple weekly use in each of the product categories).

**Table 2:** Single and multiple use (at least weekly) in each of product categories (*n* = 3,659).

	**n**	**%**
**Single use**	2206	60.3
Tobacco burning products	1462	40.0
Oral products	452	12.4
E-cigarettes	122	3.3
Cannabis products	170	4.6
Dual use	1167	31.9
**Tobacco burning products**		
+ e-cigarettes	207	5.7
+ oral products	462	12.6
+ cannabis products	414	11.3
**E-cigarettes**		
+ oral products	21	0.6
+ cannabis products	17	0.5
**Oral products**		
+ cannabis products	46	1.3
**Three or four product categories**	281	7.7

### 3.2. Product use and demographics

We compared use of products in each category across age, gender, and type of school always referring to “at least weekly” use (sum of “daily” and “weekly but not daily” use). As students aged, they more often used tobacco-burning products: 16.8% of those <16; 27.6% of those 16–17; 31.9% of those 18–19, and 44.3% of those >19 (*p* < 0.001). A similar trend was evident for cannabis products (7.1% of those <16, 9.8% of those 16–17, 10.2% of those 18–19, and 14.0% of those >19; *p* < 0.001). No such trend was detected for e-cigarettes or oral products (see Supplementary Figure 1).

We found differences in gender (men, women, and others) and product use, especially for oral products (Supplementary Figure 2). More than three times more men (16.5%) and “others” (14.7%) than women (4.6%) reported using oral products (*p* < 0.001). “Others” (33%) and men (32.4%) more often reported using tobacco burning products than women (26.4%; *p* < 0.001), and “others” used e-cigarettes more often (13.7%) than men or women (6.0% each; *p* < 0.001). Women were least likely to report using cannabis (7.3%); 12.1% of men used cannabis, as did 17.8% of “others” (*p* < 0.001).

Product use differed by type of school, especially for tobacco-burning products (Supplementary Figure 3). Significantly more basic vocational (35.3%) and higher vocational students (27.3%) used tobacco burning products than academic high school students (14.6%; *p* < 0.001). Basic vocational (12.2%) and higher vocational students (11.1%) also reported using more oral products than did high school students (6.3%; *p* < 0.001). More vocational students (6.8%) and higher vocational students (5.6%) used e-cigarettes than high school students (4.6%; *p* = 0.002). We detected no significant differences by education for cannabis products.

## 4. Discussion

### 4.1. Tobacco and nicotine products

Adolescents in Switzerland use a broad array of tobacco, nicotine, and cannabis products. By far the most common are commercially packaged tobacco cigarettes (14.2% daily use), but students regularly consume many other products. Because ours was the first Swiss study to detail use prevalence of most available tobacco and nicotine products among 15–21-year-olds, we can only speculate about use trends. Our results align with earlier studies from Switzerland, Germany, and Denmark that found adolescents were more likely to smoke tobacco cigarettes than use any other products (13–17). While the 2017 Swiss National Health Survey (SNHS) found 10.7% of Swiss youth smoked daily, we found smoking prevalence was higher (14.2%). This discrepancy may be explained by our different methodological approaches (self-reported consumption vs. interview-based results), difference in sample sizes and selection, or actual changes in consumption habits over time (8, 18, 19).

Our results suggest that adolescent consumption of cigarettes in Switzerland is higher than in Canada, the US, England, and Denmark. Prevalence of monthly cigarette smoking in Switzerland (32.1%) was higher than was for adolescents in Canada (9.3%), England (14.8%), or the US (7.9%) (8) and our reported prevalence of general use of any tobacco or nicotine product in the last 30 days was also higher. East et al. (8) did identify an overall increase in tobacco and nicotine use among 16–19-year-olds in the US, England, and Canada between 2017 and 2019, so the trend of declining cigarette use and increasing use of newer products (especially e-cigarettes) may have continued. While we found e-cigarette use in the last 30 days comparable to that in England, use in Switzerland was lower than in Canada and the US. In Switzerland, smokeless tobacco products and hookahs were used at a much higher rate than in any of these other countries, so the pattern of tobacco and nicotine product 30-day use among Swiss adolescents differed markedly from that of their age-group peers in Canada, the US, and England. In Denmark, cigarette consumption among 15–24-year-olds is significantly lower than in Switzerland, as is occasional use (weekly or less than once a week) of e-cigarettes and smokeless tobacco products. Only reported daily prevalence of e-cigarettes and smokeless tobacco is similar in Switzerland and Denmark (14).

Higher prevalence of adolescent use in Switzerland might be due to weak policy regulations against tobacco and nicotine products in Switzerland (20). An international tobacco lobby index based on 20 indicators, the Global Tobacco Industry Interference Index (GTIII) gives Switzerland a score of 92 out of a possible 100 points (higher scores indicate the tobacco industry exerts more influence), ranking Switzerland 79th of 80 countries in 2021. Strong tobacco control policy regulations likely reduce prevalence. For example, the UK scored 32 points on the GTIII, Canada 53 points, Germany 68 points, and the US 76 points.

### 4.2. Cannabis

In Europe, about 10% of 15–24-years old used cannabis within the last month (21) and the 2017 Swiss national health-survey found similar prevalence (6), but a study of 2010 data from Zurich, the largest city in Switzerland, found 23.6% of 15–19-year-olds used cannabis. The results of this latter study are much closer to the 20% prevalence we identified (22). Like our study, the Zurich study anonymously collected data in classroom settings, where students may have been more likely to make accurate reports. We believe the European drug report and Swiss national health study underestimated cannabis use among Swiss youth for two reasons. The first is that sampling bias may be due to their relatively small sample sizes, and the second is that students may be more likely to admit to using illegal substances in an anonymous online survey than during a phone survey or on a hand-written survey collected by an authority figure. If our finding of higher prevalence is accurate, this suggests current cannabis use prevention efforts are insufficient and that we need to develop new approaches to reduce use prevalence.

### 4.3. Stratified analyses by age, gender, and education

Like other studies, we found use of tobacco burning products increases with age (13, 14, 23). Neurobiological studies indicate that younger people are more quickly addicted to substances (24), and in Switzerland youth may legally smoke tobacco at 16, while in most other countries the legal smoking age is 18 (25). Raising the legal smoking age may reduce smoking prevalence in adolescents.

We found large gender differences in oral tobacco and nicotine product use. Men reported using oral products more often than women, though smoking patterns were similar (14, 26). It is possible that social representations of masculinity, stimulus-seeking, and the practices of celebrity athletes (e.g, use of snus by many hockey players) contribute to difference in use between genders (26).

Our results confirm prior findings that people with a lower level of school education smoke more cigarettes (17, 19). We found a similar but weaker tendency in e-cigarette and oral product us, suggesting prevention efforts could be targeted toward students who are unlikely to enter the higher vocational or academic track.

### 4.4. Single and multiple use

A recent study on single, dual, and triple use of cigarettes, e-cigarettes and snus in the Nordic countries found that single use was more common than multiple use. We found that tobacco burning products were more often used alone than in combination with e-cigarettes and that tobacco burning products were most often combined with cannabis, confirming prior findings that tobacco and cannabis products were often used together, while fewer users consumed only cannabis (27). The strong association between cannabis and tobacco use should be considered in the Swiss government's plans to legalize cannabis, since legalization might have an adverse effect on tobacco control and prevention.

### 4.5. Strengths and limitations

Our large sample, which exceeded national surveys, and its close match to the gender and education in the general population of adolescents in Switzerland (28, 29) strengthened our study. The response rate of 29.2% is based on the total students in all schools in the surveyed areas of two Swiss cantons. Whether the survey was passed on to the students at a certain school was depending on the person we contacted, or the individual teachers. Our study was limited by its reliance on students' self-reported data and some students may have over- or under-reported the frequency of their substance use.

## 5. Conclusion

Students who use tobacco are still most likely to smoke cigarettes, but many also use new tobacco, nicotine, and cannabis products. Use of tobacco, nicotine, and cannabis products was more common among Swiss adolescents than earlier studies suggested, and more prevalent in Switzerland than in most other high-income countries. Tobacco control policies used in other countries that have reduced smoking prevalence may also be effective in Switzerland. Prevention programs can also be tailored to meet the needs of adolescent populations with high smoking prevalence, and specifically targeted to age group, gender, and education. The substance use habits of Swiss adolescents shift rapidly as new products appear on the market and legislation is updated, so we strongly suggest that surveys that track changes in use and popularity of tobacco, nicotine, and cannabis products be administered regularly.

## Data availability statement

The raw data supporting the conclusion of this article are available upon request by email.

## Author contributions

JA, ER, MP, RA, and JJ designed the study and questionnaires. JA and ER gathered the data. JA, ER, MP, and JJ analyzed the data. JA, ER, MP, KT, RA, and JJ drafted and reviewed the manuscript and gave their final consent to submit the current form of the manuscript. All authors contributed to the article and approved the submitted version.
