# Erratum: Use of tobacco, nicotine and cannabis products among students in Switzerland

**DOI:** 10.3389/fpubh.2023.1212598

**Published:** 2023-06-08

**Authors:** Frontiers Production Office

**Affiliations:** Frontiers Media SA, Lausanne, Switzerland

**Keywords:** tobacco, nicotine, e-cigarettes, snus, cannabis, adolescent, prevalence

Due to a production error, the funder University of Bern was erroneously omitted. Statement to be added, “Open access funding by University of Bern.” The corrected Funding statement appears below:

“Data collection and publication was supported financially by the canton of Aargau. Internal funding of research time. JJ work in tobacco prevention is funded by a career development award (CDA) funded by Swiss Academy of Medical Sciences (SAMS) (YTCR-34/19). Open access funding by University of Bern.”

The publisher apologizes for this mistake. The original article has been updated.

