# Supplementary material for: Use of tobacco, nicotine and cannabis products among students in Switzerland
Source: Front Public Health. 2023 Mar 29;11:1076217. doi: 10.3389/fpubh.2023.1076217 (PMC10137165; doi:10.3389/fpubh.2023.1076217)
Supplement: Supplementary file 1 [file Data_Sheet_1.pdf]

Use of tobacco, nicotine and cannabis products among students in Switzerland

Consumption Survey

Q1: Which gender do you identify with?

- Female
- Male
- Others

Q2: Which function do you have at school?

Forward to question depending on answer:
Student at high school --> Q3
Student at 10th grade --> Q10
Student at (high) vocational school --> Q4
Teacher or employee --> Q9

- Student at high school
- Student at high vocational school
- Student at vocational school
- Student at 10th grade
- Teacher or employee

Q3: Which high school do you attend?

Forward to Q10 (regardless of the answer)

- Alte Kantonsschule Aarau
- Neue Kantonsschule Aarau
- Kantonsschule Baden
- Kantonsschule Wettingen
- Kantonsschule Wohlen
- Kantonsschule Zofingen

Q4: in which canton do you attend the (high) vocational school?

Forward to question depending on answer:
Aargau --> Q5
St. Gallen --> Q8

- Aargau
- St. Gallen

Q5: Which (high) vocational school in the canton of Aargau do you attend?

Forward to question depending on answer:
Berufsschule Gesundheit und Soziales Brugg --> Q6
other answers --> Q10

- Berufsbildungszentrum Freiamt (Lenzburg)
- Berufsbildungszentrum Fricktal
- Berufs- und Weiterbildung Zofingen (BZZ)
- Berufsschule BBB
- Berufsschule Gesundheit und Soziales Brugg
- Berufsschule Aarau
- Berufsschule Lenzburg
- BWZ Brugg Technik und Natur
- Handelsschule KV Aarau
- Landwirtschaftliches Zentrum Liebegg
- Schule für Gestaltung Aargau - Medien, Print, Design
- Zentrum Bildung - Wirtschaftsschule KV Aargau Ost

Q6: The BFGS school grounds have been declared smoke-free since summer 2020. What is your opinion on this?

Forward to Q7 (regardless of the answer)

- 1 (left) = bad idea, 5 (right) = good idea

Q7: The BFGS school grounds have been declared smoke-free since summer 2020. What is your opinion on this?

Forward to Q11 (regardless of the answer)

- 1 (left) = bad idea, 5 (right) = good idea

Q8: Which (high) vocational school in the canton of St. Gallen do you attend?

Forward to Q10 (regardless of the answer)

- Gewerbliches Berufs- und Weiterbildungszentrum St.Gallen
- Kaufmännisches Berufs- und Weiterbildungszentrum St.Gallen
- Berufs- und Weiterbildungszentrum Rorschach-Rheintal
- Berufs- und Weiterbildungszentrum Buchs
- Berufs- und Weiterbildungszentrum Sarganserland
- Berufs- und Weiterbildungszentrum Rapperswil-Jona
- Berufs- und Weiterbildungszentrum Toggenburg
- Berufs- und Weiterbildungszentrum Wil-Uzwil

Q9: At which school do you work?

Forward to question depending on answer:
AG - Berufsschule Gesundheit und Soziales Brugg --> Q7
other answers --> Q11

- AG - Berufsbildungszentrum Freiamt (Lenzburg)
- AG - Berufsbildungszentrum Fricktal
- AG - Berufs- und Weiterbildungszentrum Zofingen (BZZ)
- AG - Berufsfachschule BBB
- AG - Berufsfachschule Gesundheit und Soziales Brugg
- AG - Berufsfachschule Aarau
- AG - Berufsfachschule Lenzburg
- AG - BZW Brugg Technik und Natur
- AG - Handelsschule KV Aarau
- AG - Landwirtschaftliches Zentrum Liebegg
- AG - Schule für Gestaltung Aargau - Medien, Print, Design
- AG - Zentrum Bildung - Wirtschaftsschule KV Aargau Ost
- AG - Alte Kantonsschule Aarau
- AG - Neue Kantonsschule Aarau
- AG - Kantonsschule Baden
- AG - Kantonsschule Wettingen
- AG - Kantonsschule Wohlen
- AG - Kantonsschule Zofingen
- SG - Gewerbliches Berufs- und Weiterbildungszentrum St.Gallen
- SG - Kaufmännisches Berufs- und Weiterbildungszentrum St.Gallen
- SG - Berufs- und Weiterbildungszentrum Rorschach-Rheintal
- SG - Berufs- und Weiterbildungszentrum Buchs
- SG - Berufs- und Weiterbildungszentrum Sarganserland
- SG - Berufs- und Weiterbildungszentrum Rapperswil-Jona
- SG - Berufs- und Weiterbildungszentrum Toggenburg
- SG - Berufs- und Weiterbildungszentrum Wil-Uzwil

Q10: How old are you?

- Younger than 16 years
- 16-17 years
- 18-19 years
- Older than 19 years

Q11: Think back to the last month. How often have you used the following products in the last month?

	Never / Never in the last month	Less than once a week	At least once a week but not daily	Daily	I'd rather not say
Tobacco cigarettes in commercial packages					
Self-rolled tobacco cigarettes					
Pipes, cigars and cigarillos					
E-cigarettes with nicotine (without tobacco, incl. e-shisha, puff bar)					
E-cigarettes without nicotine (without tobacco)					
Tobacco heating products (IQOS, Ploom)					
Hookahs (e.g. shisha, narghile)					
Snus with tobacco (e.g. EPOK)					
Snus without tobacco (Nicotine Pouches e.g. LYFT, White Fox, Nordic Spirit)					
Snuff					
Cannabis smoking (with tobacco)					
Cannabis smoking (without tobacco)					
Cannabis vaping (e.g. e-joints)					

Q12: Regarding the effect on the body, risks, regulations in Switzerland - How well informed do you feel? (1: not informed at all / 10: very well informed)

- Cigarettes (1-10)
- Hookah (1-10)
- E-Cigarettes (1-10)
- Tobacco heating products (1-10)
- Snus (1-10)
- Snuff (1-10)
- Cannabis products (1-10)

Q13: Would you like to receive more information about the following products (e.g. effect on the body, risks, regulations in Switzerland)?

- Cigarettes: yes / no
- Hookah: yes / no
- E-Cigarettes: yes / no
- Tobacco heating products: yes / no
- Snus: yes / no
- Snuff: yes / no
- Cannabis products: yes / no

Q14: Do you have a comment on the survey or is there anything else you would like to say?
