# Supplementary figures and images for: Use of tobacco, nicotine and cannabis products among students in Switzerland
Source: Front Public Health. 2023 Mar 29;11:1076217. doi: 10.3389/fpubh.2023.1076217 (PMC10137165; doi:10.3389/fpubh.2023.1076217)

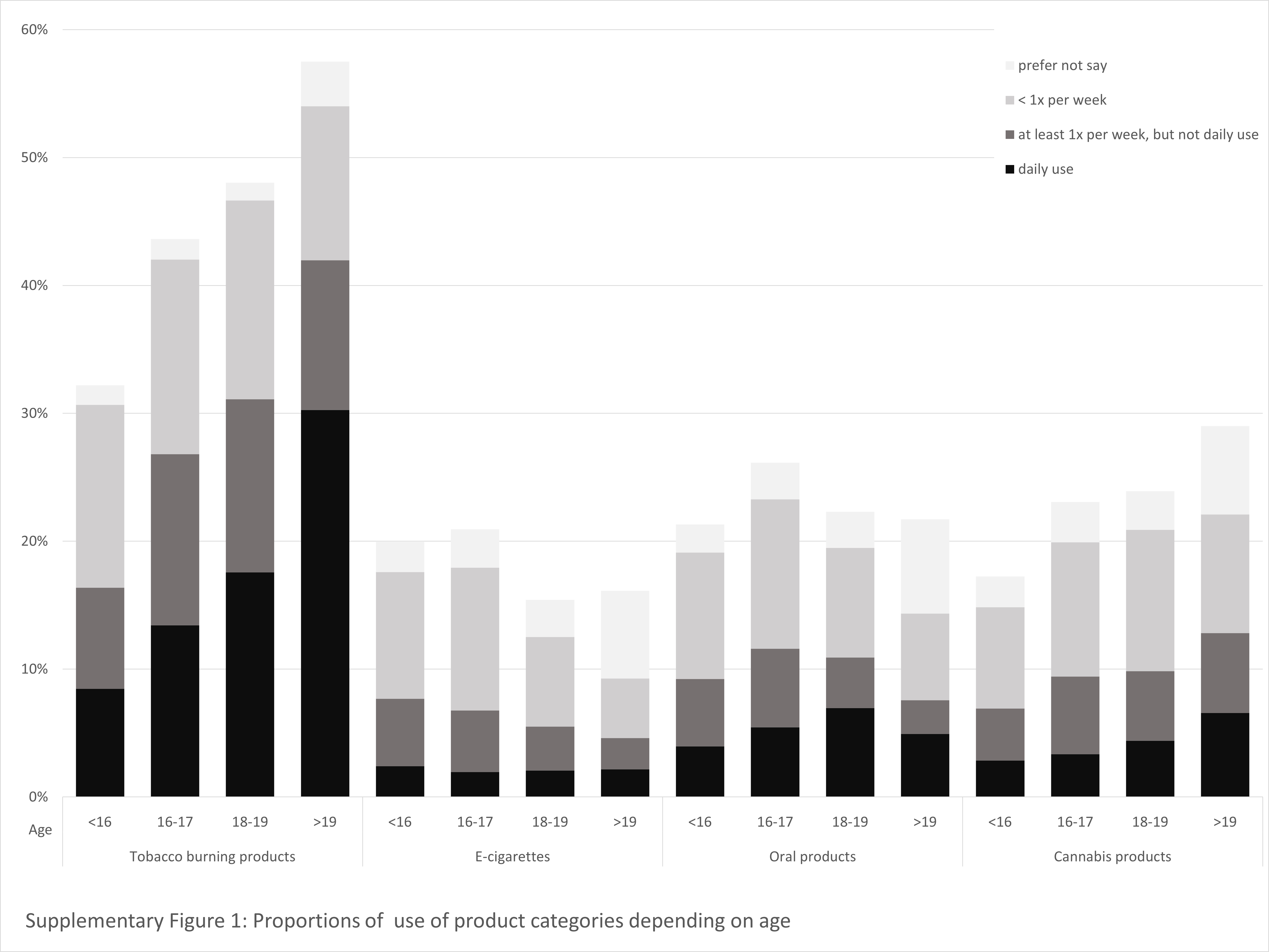

Supplement: Supplementary file 2 [file Image_1.png]

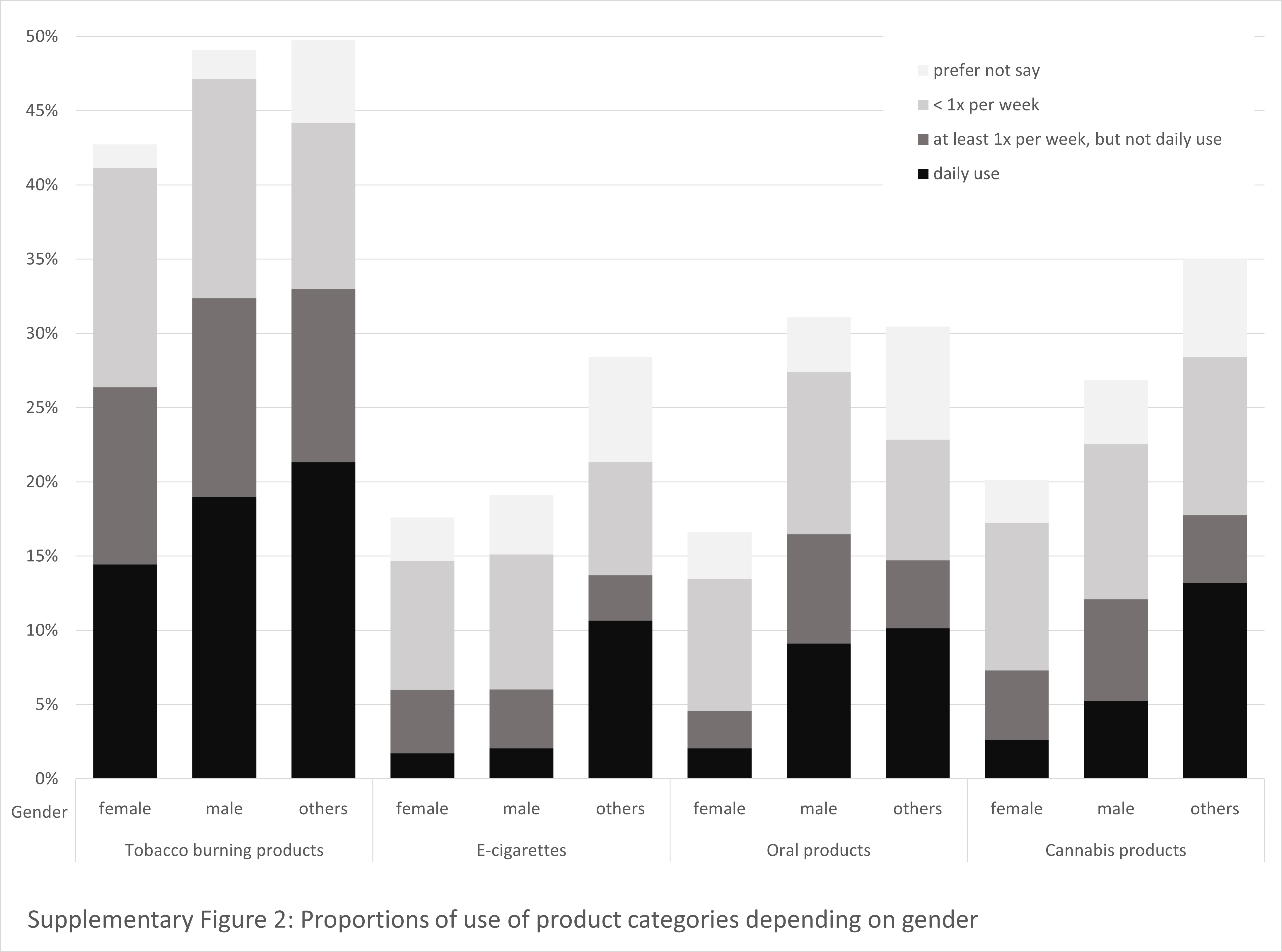

Supplement: Supplementary file 3 [file Image_2.png]

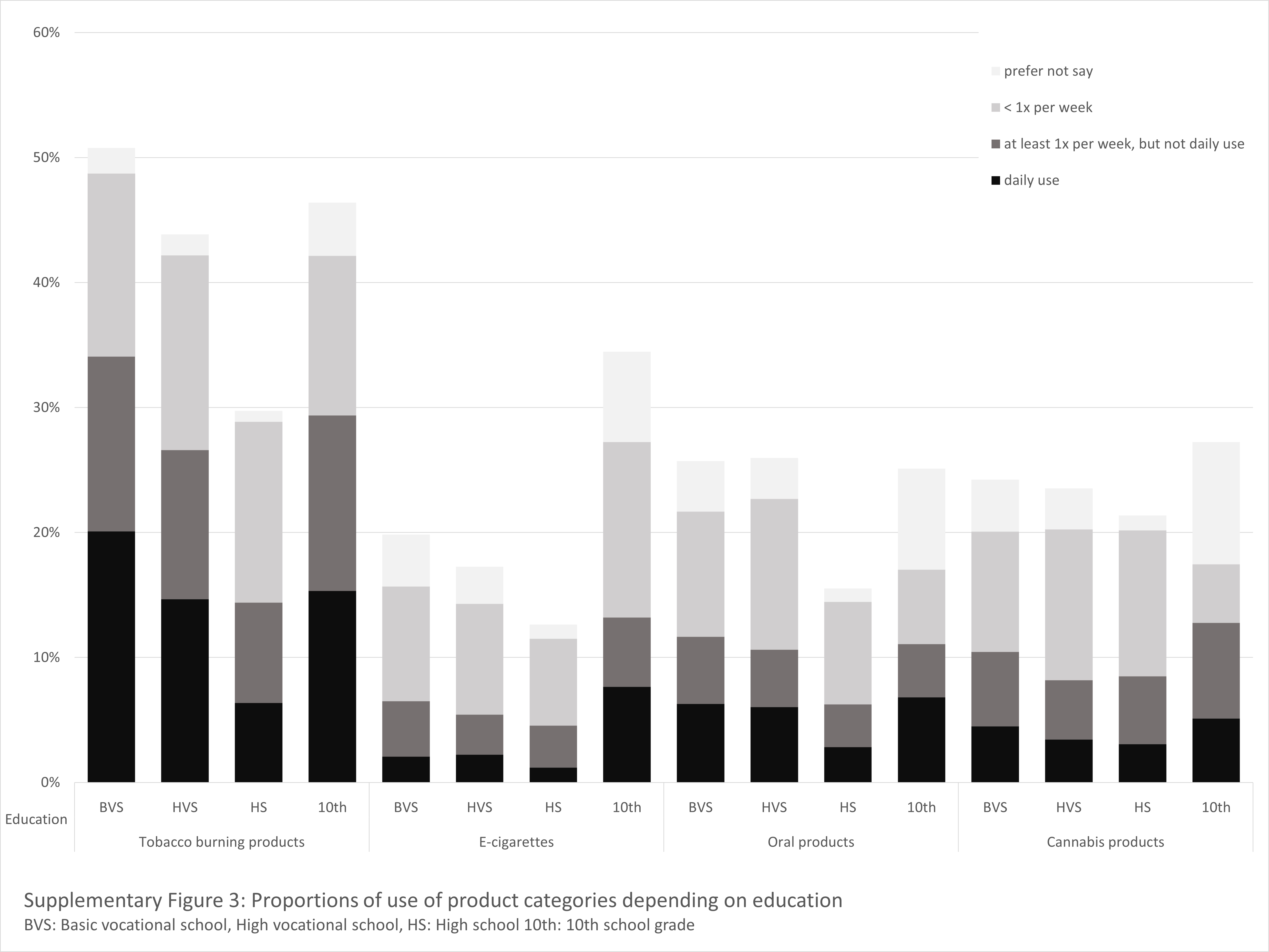

Supplement: Supplementary file 4 [file Image_3.png]
